# Estimating the Lifetime Benefits of Treatments for Heart Failure

**DOI:** 10.1016/j.jchf.2020.08.004

**Published:** 2020-12

**Authors:** João Pedro Ferreira, Kieran F. Docherty, Susan Stienen, Pardeep S. Jhund, Brian L. Claggett, Scott D. Solomon, Mark C. Petrie, John Gregson, Stuart J. Pocock, Faiez Zannad, John J.V. McMurray

**Affiliations:** a BHF Cardiovascular Research Centre, University of Glasgow, Glasgow, United Kingdom; b National Institute of Health and Medical Research (INSERM), Center for Clinical Multidisciplinary Research 1433, INSERM U1116, University of Lorraine, Regional University Hospital of Nancy, French Clinical Research Infrastructure Network (F-CRIN) Investigation Network Initiative-Cardiovascular and Renal Clinical Trialists (INI-CRCT), Nancy, France; c Heart Center, Department of Clinical and Experimental Cardiology, Amsterdam Cardiovascular Sciences. Amsterdam University Medical Center, University of Amsterdam, Amsterdam the Netherlands; d Cardiovascular Division, Brigham and Women’s Hospital, Boston, Massachusetts, USA; e Department of Biostatistics, London School of Hygiene and Tropical Medicine, London, United Kingdom

**Keywords:** restricted mean survival time, survival models, treatment effects, trials, CI, confidence interval, HF, heart failure, HFrEF, heart failure with reduced ejection fraction, HR, hazard ratio, NNT, number needed to treat, NT-proBNP, N-terminal pro–B-type natriuretic peptide, NYHA, New York Heart Association, RMST, restricted mean survival time

## Abstract

**Objectives:**

This study compared ways of describing treatment effects. The objective was to better explain to clinicians and patients what they might expect from a given treatment, not only in terms of relative and absolute risk reduction, but also in projections of long-term survival.

**Background:**

The restricted mean survival time (RMST) can be used to estimate of long-term survival, providing a complementary approach to more conventional metrics (e.g., absolute and relative risk), which may suggest greater benefits of therapy in high-risk patients compared with low-risk patients.

**Methods:**

Relative and absolute risk, as well as the RMST, were calculated in heart failure with reduced ejection fraction (HFrEF) trials.

**Results:**

As examples, in the RALES trial (more severe HFrEF), the treatment effect metrics for spironolactone versus placebo on heart failure hospitalization and/or cardiovascular death were a hazard ratio (HR) of 0.67 (95% confidence interval [CI]: 0.5 to 0.77), number needed to treat = 9 (7 to 14), and age extension of event-free survival +1.1 years (−0.1 to + 2.3 years). The corresponding metrics for EMPHASIS-HF (eplerenone vs. placebo in less severe HFrEF) were 0.64 (0.54 to 0.75), 14 (1 to 22), and +2.9 (1.2 to 4.5). In patients in PARADIGM-HF aged younger than 65 years, the metrics for sacubitril/valsartan versus enalapril were 0.77 (95% CI: 0.68 to 0.88), 23 (15 to 44), and +1.7 (0.6 to 2.8) years; for those aged 65 years or older, the metrics were 0.83 (95% CI: 0.73 to 0.94), 29 (17 to 83), and +0.9 (0.2 to 1.6) years, which provided evidence of a greater potential life extension in younger patients. Similar observations were found for lower risk patients.

**Conclusions:**

RMST event-free (and overall) survival estimates provided a complementary means of evaluating the effect of therapy in relation to age and risk. They also provided a clinically useful metric that should be routinely reported and used to explain the potential long-term benefits of a given treatment, especially to younger and less symptomatic patients.

In randomized controlled trials, the effect of treatment is usually estimated using a time-to-first-event survival model that compares the hazard rate of the experimental treatment group (or groups) and the control group, which produces a hazard ratio (HR) and corresponding 95% confidence interval (CI) (1). By convention, if the upper 95% CI does not cross unity, the effect of treatment is considered statistically significant. Although this is the standard way of reporting the effect of treatment at medical presentations and in publications, it may exaggerate the effect of therapy (e.g., if the absolute risk reduction is small) and may not be readily interpretable by patients in terms of understanding their survival free of adverse clinical events, including death (2). Reporting the absolute treatment effect, as a percent reduction, reduction in event rate, or number needed to treat (NNT), overcomes the first of these criticisms (although NNT should be standardized for duration of follow-up). However, metrics of absolute benefit will generally look better in a high-risk than in a low-risk population, assuming the proportional risk reduction with the treatment is similar, at least in the relatively short follow-up that typifies most trials. Conversely, treatments started earlier in the course of a disease when patients are at lower risk (or even in younger patients) may have the potential to lead to greater prolongation of life. Another assessment of treatment effect that complements HR and absolute risk reduction or NNT, is the restricted mean survival time (RMST) (3,4). The RMST can be interpreted as the mean event-free survival time up to a pre-specified time point and is equivalent to the area under the Kaplan-Meier curve from the start of the study up to that point. Using age at randomization instead of time, the RMST approach allows for estimation of long-term, event-free survival that can be obtained with a specific intervention compared with a control group, across different age groups (5). The RMST provides an estimate of the effect of treatment in terms of time “free of an event,” years of life gained, or both. Such measures may be more readily interpretable and quantifiable for patients and clinicians. To better understand the use of RMST and how it compares with other conventional measures of treatment effect, we analyzed HR, NNT, and RMST in several large cardiovascular outcome trials. We also analyzed these metrics in low-risk versus high-risk subgroups to illustrate how the RMST could provide relevant information that is less dependent on the risk of patients, providing a clinically relevant long-term outlook.

## Methods

### Trials and endpoints

The incidence rate and the effect of treatment illustrated as a relative risk reduction, absolute event difference, NNT, and RMST were calculated (see Statistical Analysis section) in 4 heart failure (HF) trials: PARADIGM-HF (Angiotensin–Neprilysin Inhibition versus Enalapril in Heart Failure), RALES (The Effect of Spironolactone on Morbidity and Mortality in Patients with Severe Heart Failure), EMPHASIS-HF Eplerenone in Patients with Systolic Heart Failure and Mild Symptoms-Heart Failure), and DIG (The Effect of Digoxin on Mortality and Morbidity in Patients with Heart Failure). A brief description of each of these trials and their outcomes is provided in the Supplemental Appendix. In this study, we examined 2 outcomes: 1) the composite of time to first occurrence of either HF hospitalization or cardiovascular death (used to estimate event-free survival); and 2) all-cause mortality (used to estimate overall survival). To homogenize the estimates, all follow-up times were capped at 3 years. The RMST, using age instead of time, was performed from 60 to 80 years in all trials, except for the age subgroups in PARADIGM-HF, in which in patients younger than 65 years, the age range was 50 to 64 years and in patients aged 65 or older, the age range was 65 to 80 years.

These trials were selected because they allowed comparison of the different means of quantifying the effect of treatment, as listed previously, and how patient characteristics could influence these. Because of its large sample size, we believed that the PARADIGM-HF trial would give reasonably robust estimates of the effect of treatment in subgroups (at least for the composite hospitalization and/or death outcome). This allowed us to test the hypothesis that patient risk and patient age would influence potential gains in event-free survival and overall survival, and specifically, that these gains would be smaller in higher risk and older patients compared with that of lower risk and younger patients. We tested this hypothesis by examining patients in PARADIGM-HF with a baseline N-terminal pro–B-type natriuretic peptide (NT-proBNP) level of <1,000 pg/ml (and also below the median of 1,615 pg/ml) versus ≥1,000 pg/ml (and equal or above the median), who were in New York Heart Association (NYHA) functional class I and/or II versus class III and/or IV and age younger 65 years versus age 65 years or older. In the subgroups, we focused on event-free survival, because there was more statistical power for this outcome than for all-cause mortality.

The RALES and EMPHASIS-HF trials provided an indirect comparison of how the effects of a mineralocorticoid receptor antagonist might be modified by patient risk. RALES was a trial that enrolled a high-risk advanced HF population (all patients were in NYHA functional class III and/or IV) who received suboptimal treatment by today’s standards (e.g., 10% beta-blocker use). The EMPHASIS-HF trial randomized a lower risk, relatively well-treated patient cohort (all NYHA functional class II; 87% treated with a beta-blocker).

Because digoxin did not reduce all-cause mortality, the DIG trial was used to illustrate the effects of therapy on event-free survival versus overall survival.

All of these trials had similar patient age ranges and follow-up times.

Ethics approval was obtained for each individual trial.

### Statistical analysis

Cox proportional hazards models were used to estimate HRs and relative risk reductions. Event rates and the differences, with the respective 95% CIs were calculated using the quadratic approximation to the Poisson log-likelihood for the log-rate parameter. Event-free survival was computed using the Kaplan-Meier survivor function over the full data and compared using the log-rank test. The NNT to benefit was computed from the cause-specific cumulative incidence functions. The RSMT is a measure of average event-free survival from time 0 to a pre-specified time point (6). We computed the RMST using the within-trial follow-up time and also used age instead of time (the age-specific event rates were then estimated). These estimations were obtained by multiplying the annual conditional survival probabilities of patients included in the studies, starting from a specific age, from projections of the expected duration of event-free survival (up to a pre-specified time horizon [tau]), and were non-parametrically estimated by calculation of the area under the Kaplan-Meier survival curve (5,7). Subsequently, differences in the estimated duration of event-free survival for patients treated with active drug and treatment versus those in a placebo and/or control group were interpreted as mean years of life gained (or lost). In other words, for any given age, a survival curve was estimated that represented the survival probabilities over time for patients alive at that age and who receiving an active drug and/or treatment. A corresponding survival curve was then estimated using data from patients who were in the placebo and/or control group. From each survival curve, the average time spent before the event of interest was estimated by the area under the survival curve. Because patient age and treatment assignment were independent of one another due to randomization, the difference in the areas under the survival curve could be interpreted as the effect of treatment on time spent event free. The key statistical assumption required to obtain valid long-term survival projections based on short-term follow-up data was that a patient’s risk for a given event depended on age and treatment but not on the duration of exposure to the treatment (5,8). This meant that a 79-year-old patient who took sacubitril-valsartan since the age of 60 years (i.e., 19 years of treatment exposure) had the same assumed risk of dying by age 80 years as a 79-year-old patient who started taking sacubitril-valsartan at age 78 years (i.e., 1 year of treatment exposure). For consistency, we assessed the survival data over a maximum follow-up of 3 years and an age range from 60 to 80 years (tau = 20) in all trials. In the PARADIGM-HF trial, age projection estimates were also performed for the previously described subgroups described. The p values <0.05 were considered statistically significant. All analyses were conducted using Stata version 16 (StataCorp, College Station, Texas).

## Results

### Trials analyzed

**Figure 1:**
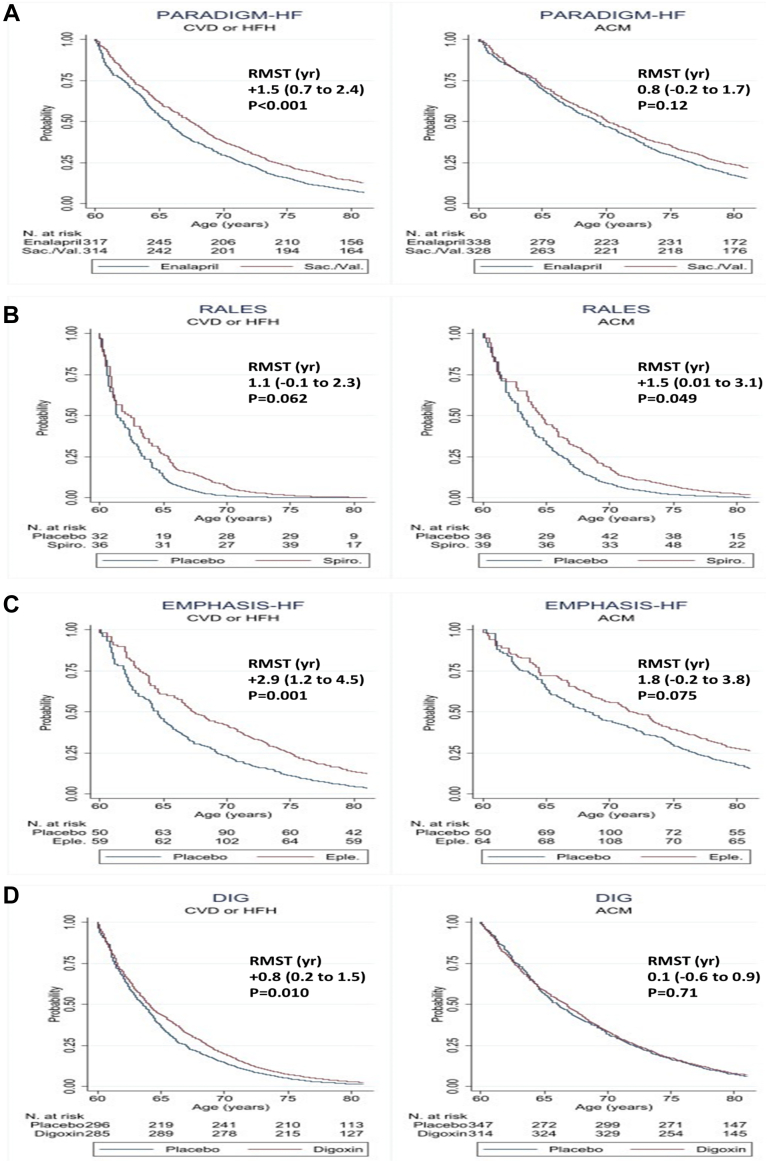
RMST Using Age Instead of Time for the Trials Included The restricted mean survival time (RMST) is expressed in potential years gained without an event from 60 to 80 years of age. The number of patients at risk represents the number of patients who were enrolled in the trial(s) in whom the event of interest had not occurred at a given age. A longer potential life extension (area under the Kaplan-Meier curve) was observed for patients at lower risk. Because they were less likely to have events or dying during follow-up, their potential life extension was improved with treatment. **(A)** PARADIGM-HF (Angiotensin–Neprilysin Inhibition versus Enalapril in Heart Failure): potential years gained without event were observed across all the age groups in patients treated with sacubitril/valsartan (vs. enalapril), especially for the composite outcome of cardiovascular death (CVD) or heart failure hospitalization (HFH). **(B)** RALES (The Effect of Spironolactone on Morbidity and Mortality in Patients with Severe Heart Failure): high-risk population, the potential years gained without event in favor of spironolactone (vs. placebo) were observed especially at younger ages within the trial. **(C)** EMPHASIS-HF (Eplerenone in Patients with Systolic Heart Failure and Mild Symptoms): a lower risk population than that in the RALES trial with a treatment effect of eplerenone (vs. placebo) on life extension that was observed across all the ages (60 to 80 years) within the trial. **(D)** DIG (The Effect of Digoxin on Mortality and Morbidity in Patients with Heart Failure): digoxin extended the time free of HFH but did not prolong life. ACM = all-cause mortality.

The overall results for each of the 4 trials analyzed are shown in Table 1 and Figure 1.

**Table 1:** Cox, Event Rates, Number Needed to Treat, Proportional Hazards, and RSMT

CV Death or HFH∗			Treatment Effect HR (95% CI)	p Value
PARADIGM-HF (N = 8,399)				
	Enalapril (n = 4,212)	Sacubitril/Valsartan (n = 4,187)		
Events	1,088 (25.8)	893 (20.0)	0.80 (0.73 to 0.88)	<0.001
Event-free survival (%)	69.1 (67.4 to 70.7)	73.7 (72.1 to 75.3)	+4.6	<0.001
NNT to benefit			25 (18 to 43)	<0.001
Event rates and difference (per 100 patient-yrs)	13.2 (12.5 to 14.1)	10.6 (9.9 to 11.3)	to 2.6 (−3.7 to −1.6)	<0.001
RMST (using follow-up time in days)	895 (884 to 906)	932 (922 to 942)	+37 (23 to 52)	<0.001
RMST (using age instead of follow-up time)†	7.3 (6.8 to 7.9)	8.9 (8.2 to 9.5)	+1.5 (0.7 to 2.4)	<0.001
All-cause death∗				
Events	796 (24.3)	683 (21.0)	0.85 (0.77 to 0.94)	0.002
Event-free survival (%)	76.2 (74.6 to 77.7)	79.4 (77.8 to 80.8)	+3.2	0.002
NNT to benefit			43 (26 to 116)	0.002
Event rates and difference (per 100 patient-yrs)	7.6 (7.1 to 8.2)	8.9 (8.3 to 9.6)	−1.3 (−2.2 to −0.1)	0.002
RMST (using follow-up time in days)	961 (952 to 970)	980 (971 to 988)	+19 (7 to 31)	0.002
RMST (using age instead of follow-up time)†	10.1 (9.5 to 10.8)	10.9 (10.2 to 11.6)	0.8 (−0.2 to 1.7)	0.12
RALES (N =1,663)				
	Placebo (n = 841)	Spironolactone (n = 822)		
Events	512 (60.9)	381 (46.4)	0.67 (0.59 to 0.77)	<0.001
Event-free survival (%)	31.9 (27.9 to 36.0)	48.0 (43.2 to 52.6)	+16.1	<0.001
NNT to benefit			9 (7 to 14)	<0.001
Event rates and difference (per 100 patient-yrs)	45.4 (41.6 to 49.5)	29.3 (26.5 to 32.5)	−16.0 (−21.0 to −11.1)	<0.001
RMST (using follow-up time in days)	574 (545 to 602)	693 (664 to 722)	+119 (78 to 160)	<0.001
RMST (using age instead of follow-up time)†	2.3 (1.7 to 3.0)	3.4 (2.5 to 4.4)	1.1 (−0.1 to 2.3)	0.062
All-cause death∗				
Events	384 (45.7)	284 (34.5)	0.71 (0.61 to 0.83)	<0.001
Event-free survival (%)	46.1 (41.5 to 50.5)	60.9 (56.5 to 65.0)	+14.8	<0.001
NNT to benefit			12 (8 to 21)	<0.001
Event rates and difference (per 100 patient-yrs)	26.6 (24.1 to 29.4)	18.8 (16.7 to 21.1)	−7.8 (−11.2 to −4.4)	<0.001
RMST (using follow-up time in days)	736 (709 to 762)	812 (786 to 837)	+76 (39 to 113)	<0.001
RMST (using age instead of follow-up time)†	4.2 (3.3 to 5.2)	5.8 (4.6 to 7.0)	+1.5 (0.01 to 3.1)	0.049
EMPHASIS-HF (N = 2,737)				
	Placebo (n = 1,371)	Eplerenone (n = 1,362)		
Events	349 (25.4)	235 (17.3)	0.64 (0.54 to 0.75)	<0.001
Event-free survival (%)	64.1 (60.5 to 67.4)	73.9 (70.5 to 77.0)	+9.8	<0.001
NNT to benefit			14 (10 to 22)	<0.001
Event rates and difference (per 100 patient-yrs)	16.7 (15.0 to 18.5)	10.5 (9.3 to 12.0)	to 6.2 (−8.3 to −4.0)	<0.001
RMST (using follow-up time in days)	827 (806 to 848)	905 (887 to 923)	+78 (51 to 106)	<0.001
RMST (using age instead of follow-up time)†	6.3 (5.3 to 7.4)	9.2 (7.9 to 10.4)	+2.9 (1.2 to 4.5)	0.001
All-cause death∗				
Events	201 (14.7)	157 (11.5)	0.76 (0.62 to 0.94)	0.010
Event-free survival (%)	77.1 (73.7 to 80.0)	81.8 (78.7 to 84.5)	+4.7	0.010
NNT to benefit			33 (19 to 142)	0.028
Event rates and difference (per 100 patient-yrs)	8.7 (7.6 to 10.0)	6.6 (5.7 to 7.7)	−2.1 (−3.7 to −0.5)	0.010
RMST (using follow-up time in days)	949 (932 to 966)	978 (963 to 994)	+29 (7 to 52)	0.010
RMST (using age instead of follow-up time)†	8.4 (6.9 to 9.9)	10.2 (8.9 to 11.6)	1.8 (−0.2 to 3.8)	0.075
DIG (N = 6,800)				
	Placebo (n = 3,403)	Digoxin (n = 3,397)		
Events	1,515 (44.5)	1,335 (39.3)	0.82 (0.76 to 0.88)	<0.001
Event free survival (%)	54.2 (52.5 to 55.9)	59.4 (57.7 to 61.0)	+5.2	<0.001
NNT to benefit			20 (15 to 32)	<0.001
Event rates and difference (per 100 patient-yrs)	22.0 (20.9 to 23.2)	18.0 (17.1 to 19.0)	−4.0 (−5.5 to −2.6)	<0.001
RMST (using follow-up time in days)	767 (753 to 781)	830 (817 to 843)	+63 (44 to 82)	<0.001
RMST (using age instead of follow-up time)†	4.9 (4.5 to 5.4)	5.8 (5.3 to 6.2)	+0.8 (0.2 to 1.5)	0.010
All-cause death∗				
Events	1,004 (29.5)	1,007 (29.6)	1.00 (0.92 to 1.09)	0.99
Event-free survival (%)	69.8 (68.2 to 71.4)	69.6 (68.0 to 71.1)	0.2	0.99
NNT to benefit			NA	—
Event rates and difference (per 100 patient-yrs)	12.0 (11.3 to 12.8)	12.1 (11.3 to 12.8)	0.0 (−1.0 to 1.0)	0.99
RMST (using follow-up time in days)	916 (905 to 927)	922 (912 to 933)	6 (−9 to 21)	0.46
RMST (using age instead of follow-up time)†	7.9 (7.3 to 8.4)	8.0 (7.5 to 8.5)	0.1 (−0.6 to 0.9)	0.71

Values are n, n (%), or median (interquartile range).

The event-free survival was computed using the Kaplan-Meier survivor function over the full data and compared using the log-rank test.

The number needed to treat (NNT) to benefit was computed from the cause-specific cumulative incidence functions.

Median (25th to 75th percentile) follow-up time (days) and age at randomization (years): PARADIGM-HF: 810 days (564 to 1,069 days) and 64 years (57 to 72 years); EMPHASIS-HF: 639 days (292 to 992 days) and 68 yrs (63 to 74 years); RALES: 714 days (381 to 909 days) and 67 years (59 to 73 years); DIG: 1,152 days (843 to 1,423 days) and 65 years (57 to 71 years).

CV = cardiovascular; DIG = Effect of Digoxin on Mortality and Morbidity in Patients with Heart Failure; EMPHASIS-HF = Eplerenone in Patients with Systolic Heart Failure and Mild Symptoms; HR = hazard ratio; PARADIGM-HF = Angiotensin–Neprilysin Inhibition versus Enalapril in Heart Failure; RALES = Effect of Spironolactone on Morbidity and Mortality in Patients with Severe Heart Failure; RMST = restricted mean survival time.

∗ For consistency, the analysis the follow-up time was capped at 3 years.

† For consistency, the RMST used age instead of time and used the same age range from 60 to 80 years in all the studied trials.

#### PARAGIGM-HF

Overall, in the PARADIGM-HF trial, 25.8% (13.2 per 100 patient-years) of the patients in the enalapril group versus 20.0% (10.6 per 100 patient-years) of the patients in the sacubitril and/or valsartan group experienced the primary composite outcome (HF hospitalization or cardiovascular death) at 3 years of follow-up, which gave an HR of 0.80 (95% CI: 0.73 to 0.88), and a NNT of 25 (18 to 43). The RMST days gained over this follow-up was +37 (23 to 52) days, and the potential extension of life without an event was estimated at +1.5 (0.7 to 2.4) years. The findings for all-cause death and an increase in overall survival are shown in Table 1 and Figure 1.

#### RALES

In the RALES study, 60.9% (45.5 per 100 patient-years) of the patients in the placebo group versus 46.4% (29.3 per 100 patient-years) of the patients in the spironolactone group experienced the primary outcome over 3 years of follow-up, with a corresponding HR of 0.67 (95% CI: 0.59 to 0.77) and NNT of 9 (7 to 14). The RMST days gained during follow-up were +119 (78 to 160) days and an extension of event-free survival of +1.1 (−0.1 to +2.3) years. The findings for all-cause death and increase in overall survival are shown in Table 1 and Figure 1.

#### EMPHASIS-HF

In the EMPHASIS-HF trial, 25.4% (16.7 per 100 patient-years) of the patients in the placebo group versus 17.3% (10.5 per 100 patient-years) of the patients in the eplerenone group experienced the primary outcome at 3 years of follow-up, which gave an HR of 0.64 (95% CI: 0.54 to 0.75) and a NNT of 14 (10 to 22). The RMST days gained were +78 (51 to 106) days and an extension of event-free survival of +2.9 (1.2 to 4.5) years. The findings for all-cause death and increase in overall survival are shown in Table 1 and Figure 1.

#### DIG

In the DIG trial, 44.5% (22.0 per 100 patient-years) of the patients in the placebo group versus 39.3% (18.0 per 100 patient-years) of the patients in the digoxin group experienced the primary outcome over 3 years of follow-up; the HR was 0.82 (95% CI: 0.76 to 0.88) in favor of digoxin, and the NNT was 20 (15 to 32). Patients who took digoxin gained +63 (44 to 82) days of follow-up and +0.8 (0.2 to 1.5) years of event-free survival. Digoxin did not reduce all-cause death because there was no increase in event-free survival (Table 1).

### Subgroups in PARADIGM-HF

**Figure 2:**
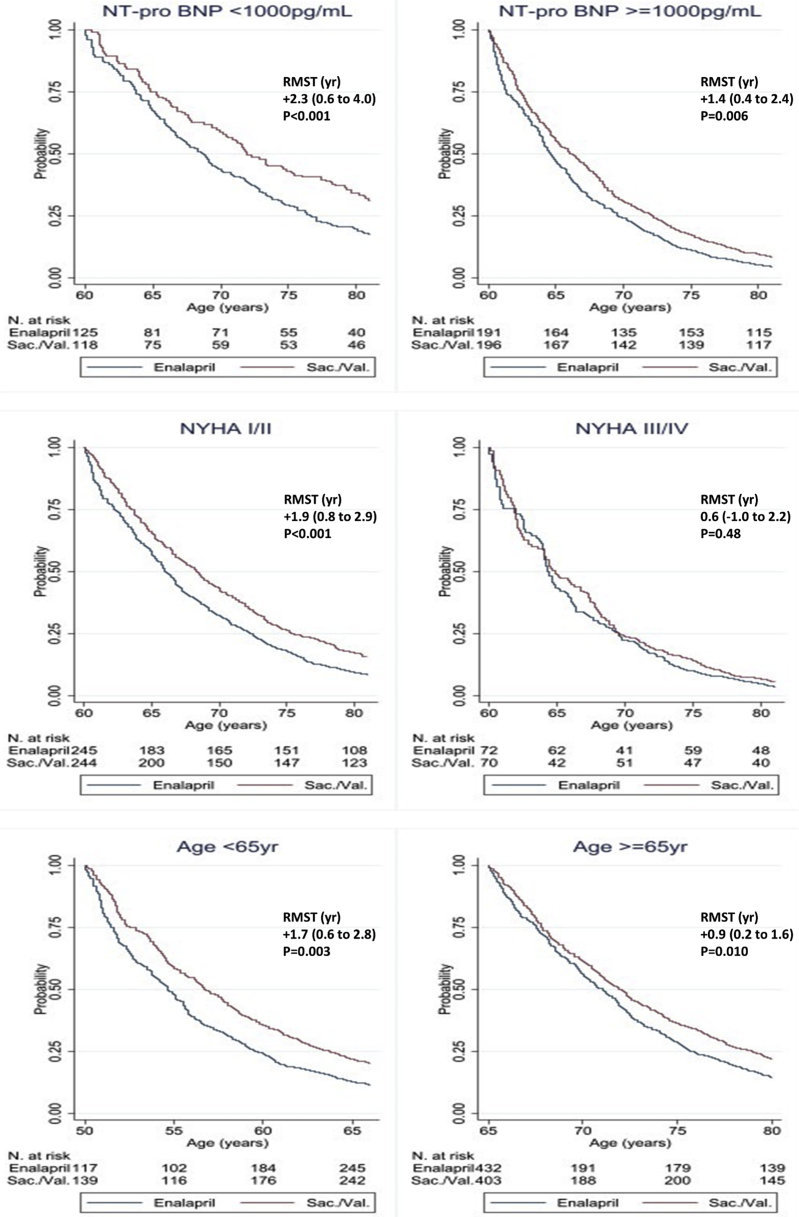
PARADIGM-HF: RMST Using Age Instead of Time in Subgroups Reflecting Patient Risk and Age The number of patients at risk represents the number of patients who were enrolled in the trial(s) in whom the event of interest had not occurred at a given age. A longer potential life extension (area under the Kaplan-Meier curve) was observed for patients with lower N-terminal pro–B-type natriuretic peptide (NT-proBNP) levels, who were in New York Heart Association (NYHA) functional class I and/or II, and were younger than 65 years, which suggested that the long-term benefits of sacubitril/valsartan were particularly important in less symptomatic and younger patients (i.e., patients with lower risk). Abbreviations as in Figure 1.

The results for each of the 3 subgroups analyzed are shown in Table 2, Figure 2, and Supplemental Table 1.

#### Lower baseline NT-proBNP level versus higher baseline NT-proBNP level

Low NT-proBNP was defined as <1,000 pg/ml and high NT-proBNP as ≥1,000 pg/ml (Table 2). The analysis was also performed using the trial median NT-pro BNP of 1, 615 pg/ml (Supplemental Table 1).

**Table 2:** Treatment Effect Estimates in PARADIGM-HF Subgroups (N = 8,399)

CV death or HFH∗	Enalapril (n = 4,212)	Sacubitril/Valsartan (n = 4,187)	Treatment Effect HR (95% CI)	p Value
NT-proBNP <1,000 pg/ml (N = 2,481)	1,262	1,219		
Events	212 (16.8)	153 (12.6)	0.73 (0.59 to 0.91)	0.004
Event-free survival (%)	79.2 (76.2 to 81.8)	84.5 (82.2 to 87.2)	+5.3	0.004
NNT to benefit			27 (16 to 83)	0.004
Event rates and difference (per 100 patient-yrs)	7.6 (6.6 to 8.7)	5.6 (4.7 to 6.5)	to 2.0 (−3.4 to −0.7)	0.004
RMST (using follow-up time in days)	979 (964 to 994)	1,005 (991 to 1,019)	+26 (6 to 47)	0.013
RMST (using age instead of follow-up time)†	9.9 (8.7 to 11.0)	12.2 (10.9 to 13.5)	+2.3 (0.6 to 4.0)	0.009
NT-proBNP ≥1,000 pg/ml) (N = 5,904)	2,941	2,963		
Events	902 (30.7)	760 (25.7)	0.81 (0.73 to 0.89)	<0.001
Event-free survival (%)	64.8 (62.7 to 66.8)	69.0 (66.8 to 71.0)	+4.2	<0.001
NNT to benefit			24 (16 to 44)	<0.001
Event rates and difference (per 100 patient-yrs)	16.0 (15.0 to 17.1)	12.9 (12.0 to 13.9)	−3.1 (−4.5 to −1.7)	<0.001
RMST (using follow-up time in days)	849 (835 to 862)	891 (879 to 903)	+42 (24 to 61)	<0.001
RMST (using age instead of follow-up time)†	6.4 (5.7 to 7.0)	7.8 (7.0 to 8.5)	+1.4 (0.4 to 2.4)	0.006
NYHA functional class I/II (N = 5,057)	2,511	2,546		
Events	759 (30.2)	597 (23.4)	0.75 (0.68 to 0.84)	<0.001
Event-free survival (%)	70.8 (68.8 to 72.6)	76.6 (74.7 to 78.3)	+5.8	<0.001
NNT to benefit			21 (15 to 34)	<0.001
Event rates and difference (per 100 patient-yrs)	12.2 (11.4 to 13.1)	9.2 (8.5 to 9.9)	−3.0 (−4.1 to −1.8)	<0.001
RMST (using follow-up time in days)	910 (898 to 922)	952 (942 to 963)	+42 (26 to 58)	<0.001
RMST (using age instead of follow-up time)†	7.8 (7.1 to 8.5)	9.6 (8.9 to 10.4)	+1.9 (0.8 to 2.9)	<0.001
NYHA functional class III/IV (N = 1,695)	892	803		
Events	329 (36.9)	295 (36.7)	0.93 (0.80 to 1.09)	0.40
Event-free survival (%)	63.9 (60.3 to 67.2)	64.7 (61.1 to 68.2)	0.8	0.40
NNT to benefit			NA	-
Event rates and difference (per 100 patient-yrs)	16.2 (14.9 to 18.5)	15.5 (13.7 to 17.3)	−1.1 (−3.6 to 1.4)	0.36
RMST (using follow-up time in days)	828 (806 to 850)	847 (825 to 869)	19 (−12 to 49)	0.24
RMST (using age instead of follow-up time)†	6.2 (5.2 to 7.3)	6.8 (5.6 to 8.0)	0.6 (−1.0 to 2.2)	0.48
Age <65 yrs (N = 4,279)	2,168	2,111		
Events	545 (25.1)	422 (20.0)	0.77 (0.68 to 0.88)	<0.001
Event-free survival (%)	70.0 (67.6 to 72.2)	75.6 (73.3 to 77.8)	+5.6	<0.001
NNT to benefit			23 (15 to 44)	<0.001
Event rates and difference (per 100 patient-yrs)	13.0 (11.9 to 14.1)	10.0 (9.1 to 11.0)	−3.0 (−4.4 to −1.5)	<0.001
RMST (using follow-up time in days)	889 (874 to 904)	930 (916 to 943)	+40 (21 to 61)	<0.001
RMST (using age instead of follow-up time)†	6.0 (5.2 to 6.7)	7.7 (6.9 to 8.4)	+1.7 (0.6 to 2.8)	0.003
Age ≥65 yrs (N = 4,120)	2,044	2,076		
Events	543 (26.6)	471 (22.7)	0.83 (0.73 to 0.94)	0.003
Event-free survival (%)	68.2 (65.7 to 70.5)	72.0 (69.5 to 74.2)	+3.8	0.001
NNT to benefit			29 (17 to 83)	0.003
Event rates and difference (per 100 patient-yrs)	13.5 (12.4 to 14.7)	11.2 (10.2 to 12.2)	−2.3 (−3.9 to −0.1)	0.001
RMST (using follow-up time in days)	892 (877 to 907)	925 (912 to 940)	+33 (13 to 54)	0.002
RMST (using age instead of follow-up time)†	6.9 (6.4 to 7.3)	7.8 (7.3 to 8.3)	+0.9 (0.2 to 1.6)	0.010

Values are n, n (%), or median (interquartile range).

The event-free survival was computed using the Kaplan-Meier survivor function over the full data and compared using the log-rank test.

The number needed to treat (NNT) to benefit was computed from the cause-specific cumulative incidence functions.

CI = confidence interval; CVD = cardiovascular death; HFH = hospitalization for heart failure; HR = hazard ratio; NA = not applicable because the absolute risk reduction is not statistically significant; NT-proBNP = N-terminal pro–B-type natriuretic peptide; NYHA = New York Heart Association.

∗ For consistency, the analysis using follow-up time was capped at 3 years.

† For consistency, the restricted mean survival time (RMST) using age instead of time used the same age range from 60 to 80 years in the studied subgroups of the PARADIGM-HF (Angiotensin–Neprilysin Inhibition versus Enalapril in Heart Failure) trial, except for the age subgroups in which in patients younger than 65 years, the age range was 50 to 64 years and in patients age 65 or older, the age range was 65 to 80 years (i.e., tau =15). This metric is expressed in years.

Among patients with a NT-pro BNP <1,000 pg/ml, the levels were 680 pg/ml (quartile 1 to quartile 3 [Q1 to Q3]: 527 to 827 pg/ml), and the mean ± SD age was 61.8 ± 11.2 years. The primary outcome was experienced in 16.8% (7.6 per 100 patient-years) of those in the enalapril group and 12.6% (5.6 per 100 patient-years) in the sacubitril/valsartan group over 3 years of follow-up, which gave a HR of 0.73 (95% CI: 0.59 to 0.91), a NNT of 27 (16 to 83), and +26 (6 to 47) days of RSMT gained during follow-up. The estimated extension of event-free survival was +2.3 (0.6 to 4.0) years.

Among patients with a NT-proBNP ≥1,000 pg/ml, the levels were 2,330 pg/ml (Q1 to Q3: 1,512 to 4,361 pg/ml), and mean age was 64.6 ± 11.4 years. Among these patients, the event rates were higher (30.7%; 16.0 per 100 patient-years in the enalapril group vs. 25.7%; 12.9 per 100 patient-years in the sacubitril/valsartan group), and the relative risk reduction was smaller (HR: 0.81; 95% CI: 0.73 to 0.89), but there was a lower NNT of 24 (16 to 44). RMST days gained during the follow-up were similar +42 (24 to 61) days, and the estimated extension of event-free survival was smaller, at +1.4 (0.4 to 2.4) years.

Among patients with NT-proBNP below the median, the levels were 888 pg/ml (Q1 to Q3: 642 to 1,201 pg/ml), and the mean age was 62.8 ± 11.1 years. These patients experienced the primary outcome in 18.2% (8.8 per 100 patient-years) of those in the enalapril group and in 13.9% (6.5 per 100 patient-years) in the sacubitril/valsartan group over 3 years of follow-up, which gave an HR of 0.74 (95% CI: 0.63 to 0.86), a NNT of 24 (16 to 48), and +34 (17 to 51) days of RSMT gained during follow-up. The estimated extension of event-free survival was +2.4 (1.1 to 3.8) years.

Among patients with a NT-pro BNP above the median, the levels (Q1, Q3) were 3,231 (2,186, 5,593) pg/ml; and mean (SD) age was 64.8 (11.6) years. Among these individuals, the event rates were higher (33.4%, 18.4 per 100 patient-years in the enalapril group vs. 28.7%, 15.2 per 100 patient-years in the sacubitril/valsartan group). The relative risk reduction was smaller (HR: 0.83; 95% CI: 0.75 to 0.93), but there was a similar NNT of 25 (16 to 61). RMST days gained during the follow-up were similar at +41 (18 to 63) days, and the estimated extension of event-free survival was smaller 0.9 (−0.1 to 2.0) years.

#### Patients in NYHA functional class I/II versus NYHA functional class III/IV

Among individuals in NYHA functional class I and/or II (mean age: 63.1 ± 11.5 years), 30.2% (12.2 per 100 patient-years) of those in the enalapril group and 23.4% (9.2 per 100 patient-years) of patients in the sacubitril/valsartan group experienced the primary outcome over 3 years of follow-up, which gave an HR of 0.75 (95% CI: 0.68 to 0.84), a NNT of 21 (15 to 34), and +42 (26 to 58) days of RSMT gained during follow-up. The estimated extension of event-free survival was +1.9 (0.8 to 2.9) years.

In patients in NYHA functional class III and/or IV (mean age: 66.0 ± 10.6 years), the event rates were higher (36.9%, 16.2 per 100 patient-years in the enalapril group vs 36.7%, 15.5 per 100 patient-years in the sacubitril/valsartan group), and the relative risk reduction was smaller (HR: 0.93; 95% CI: 0.80 to 1.09). The RMST days gained during the follow-up were 19 (−12 to 49) days, and the estimated extension of event-free survival was 0.6 (−1.0 to 2.2) years, both of which were nonsignificant and of smaller magnitude than in the NYHA functional class I and/or II group.

### Younger patients versus older patients

Among individuals aged younger than 65 years (mean age: 54.9 ± 8.0 years), 25.1% (13.0 per 100 patient-years) of those in the enalapril group and 20.0% (10.0 per 100 patient-years) of patients in the sacubitril/valsartan group experienced the primary outcome over 3 years of follow-up, which gave an HR of 0.77 (95% CI: 0.68 to 0.88), a NNT of 23 (15 to 44) and +40 (21 to 61 days) of RSMT gained during follow-up. The estimated extension of event-free survival was +1.7 (0.6 to 2.8) years.

In patients aged 65 years or older (mean age: 73.0 ± 5.7 years), the event rates were higher (26.6%; 13.5 per 100 patient-years in the enalapril group vs. 22.7%; 11.2 per 100 patient-years in the sacubitril/valsartan group), the relative risk reduction was smaller (HR: 0.83; 95% CI: 0.73 to 0.94), and the NNT was larger at 29 (17 to 83). RMST days gained during the follow-up was similar +33 (13 to 54) days, and the estimated extension of event-free survival was smaller +0.9 (0.2 to 1.6) years.

The summary of the main characteristics of the RMST compared with absolute and relative risk metrics is provided in the Central Illustration.

**Central Illustration:**
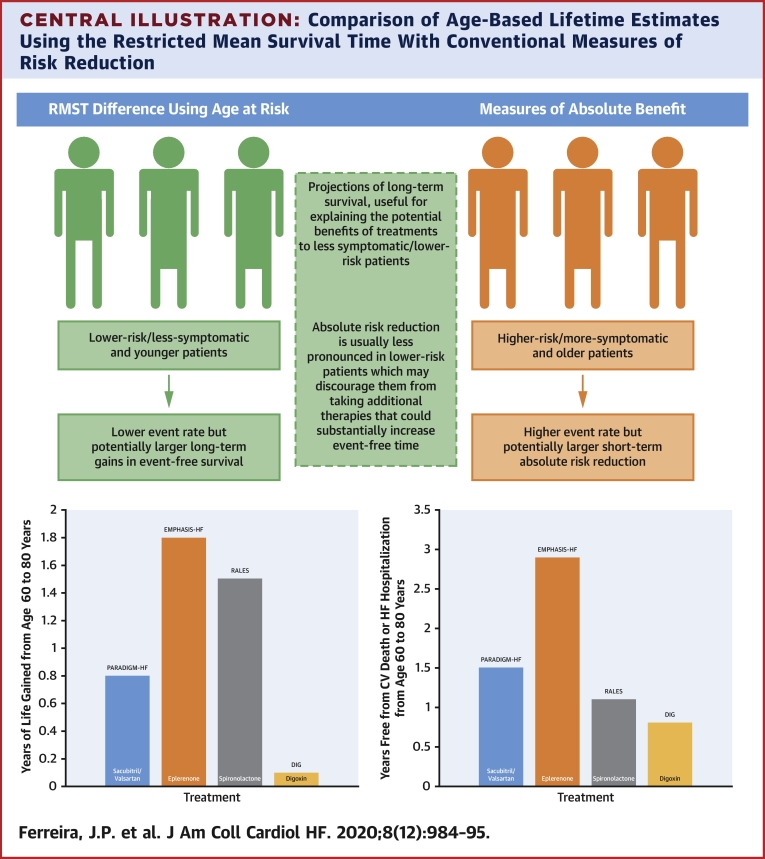
Comparison of Age-Based Lifetime Estimates Using the Restricted Mean Survival Time With Conventional Measures of Risk Reduction The restricted mean survival time (RMST) using age instead of time allows the estimation of long-term, event-free survival, which is a clinically meaningful metric for both the clinicians and patients. The projections of long-term survival may be particularly useful for explaining the potential long-term benefits of treatments to less symptomatic/lower risk patients. In contrast, the absolute risk reduction may be less pronounced in lower risk patients, which may discourage them from taking additional therapies that could substantially increase the long-term event-free time. DIG = The Effect of Digoxin on Mortality and Morbidity in Patients with Heart Failure; EMPHASIS-HF = Eplerenone in Patients with Systolic Heart Failure and Mild Symptoms; Eple. = eplerenone; HF = heart failure; HR = hazard ratio; PARADIGM-HF = Angiotensin–Neprilysin Inhibition versus Enalapril in Heart Failure; RALES = The Effect of Spironolactone on Morbidity and Mortality in Patients with Severe Heart Failure Sac./Val. = sacubitril/valsartan; Spiro. = spironolactone.

## Discussion

In this study, we showed how analysis of RMST can complement conventional ways of describing the benefit of treatment in clinical trials, which expanded on our previous descriptions of using this metric (5,9). We chose several exemplar trials to illustrate the strengths and limitations of the different ways of describing the effect of treatment. The absolute treatment benefit, whether expressed as a percent reduction, rate reduction, or NNT, was greatly influenced by the absolute risk of the patients studied, assuming the proportional risk reduction with treatment was similar across all populations. The absolute risk also reflected use of effective background therapy. We illustrated this in 2 ways. First, by comparison of patients in the PARADIGM-HF trial with higher NT-proBNP versus lower NT-proBNP (or NYHA functional class I and II vs NYHA functional class III and IV), in which patients with less severe or advanced HF had 2 to 3 times longer event-free survival than patients with more severe or advanced HF. Comparison of overall survival in the RALES and EMPHASIS-HF trials supported these findings, with the sicker, less well-treated patients in the RALES study having a NNT at 3 years to prevent 1 death of only 12 patients, compared with 33 patients in the EMPHASIS-HF trial, despite similar relative risk reductions of 0.71 (0.61 to 0.83) in the RALES study and 0.76 (0.62 to 0.94) in the EMPHASIS-HF trial. Yet, the potential years of life gained in the RALES study was 1.5 years (0.01 to 3.1 years) compared with 1.8 years (0.2 to 3.8 years) in the EMPHASIS-HF trial, with an even bigger difference in event-free survival: 1.1 years (−0.1 to 2.3 years) versus 2.9 years (1.2 to 4.5 years). This was probably because the ability to extend the duration of life in very sick patients, with any treatment, was limited. This was also the case in older patients compared with younger patients. The latter point was illustrated by our analysis of the PARADIGM-HF trial, which examined extension of life over a 15-year follow-up period. In patients who started treatment before the age of 65 years, the gain in event-free survival was 1.7 years, compared with 0.9 years in those who started treatment at aged 65 years or older. We previously illustrated this for other ages in the PARADIGM-HF and EMPHASIS-HF studies (5,9).

Consequently, age and risk (which, in part, is related to age) are both important considerations when evaluating treatment benefit. In an extreme case, a treatment might postpone or prevent many premature events in the short term (i.e., the duration of a typical trial) but lead to relatively little life-extension in a very sick, older adult population. Conversely, the same treatment could result in a much more modest, short-term absolute risk reduction in less sick, younger patients, yet lead to a substantial extension in length of life. In both trials, the relative risk reduction might be the same. These examples illustrated how RMST might be useful in discussions with payers about the use of treatments in younger or lower risk patients in whom conventional metrics of treatment benefit (absolute risk reduction, NNT) might not look favorable. They might also be useful in patients who might be reluctant to consider embarking on treatment at a young age (and potentially facing many years of treatment) or adding another treatment to several that they might already be taking.

This is not to diminish the importance of delaying or preventing nonfatal events and prolonging event-free survival, which is possible even with treatments that do not alter all-cause mortality. This was illustrated by our analysis of the DIG trial, in which the gain in event-free survival was due to a reduction in hospitalizations for HF but not mortality.

### Study limitations

These were post hoc analyses. Our findings were derived from trial data, and their generalizability to a real-world population might be limited. Subgroup analyses might not always provide robust estimates of the true effect of a treatment. Although we used NT-proBNP and NYHA functional class as proxies for risk, risk was a multivariable construct. The use of the RMST with age instead of follow-up time required a wide range of age in the population analyzed and a sufficiently large number of events across the age spectrum to provide relatively stable age-specific risk estimates. The proposed method made some major statistical assumptions and was therefore only suitable for the exploratory analyses in this study. The key assumption was that although a patient's risk of an event was related to their age and treatment group, it was not related to the length of time they spent in the study. Therefore, this methodology would not be suitable in studies in which the event rate was substantially elevated in the period shortly after randomization (e.g., in surgical trials or trials with a large variation in underlying patient risk). The proposed method would also be unsuitable in the presence of a competing risk (e.g., non-cardiovascular death) that was either frequent or imbalanced between treatment groups.

## Conclusions

RMST event-free (and overall) survival estimates provide a complementary means of evaluating the effect of therapy in relation to age and risk. It provides a clinically useful metric that should be routinely reported and used to explain the potential long-term benefits of a given treatment, especially to younger and less symptomatic patients who might be more reluctant to adhere to a treatment for life.

Perspectives **COMPETENCY IN MEDICAL KNOWLEDGE:** This work provided relevant information on the use of RMST, using follow-up time and age instead of time as a complement to the treatment effect estimates in HF trials. RMST provided an additional means of evaluating the effect of therapy in relation to age and risk, and provided a clinically useful metric that should be routinely reported and used to explain the potential long-term benefits of a given treatment, especially to younger and less symptomatic patients who might be more reluctant to adhere to a treatment for life.

**TRANSLATIONAL OUTLOOK:** This work might help change the landscape of how trial results are presented and help in the discussions with payers, clinicians, and patients.

## Author Disclosures

Drs. Ferreira and Zannad are supported by ANR-15-RHU-0004 and ANR-15-IDEX-04-LUE. Drs. Jhund, Petrie, and McMurray are supported by the British Heart Foundation (grant RE/18/6/34217). All other authors have reported that they have no relationships relevant to the contents of this paper to disclose.

## References

[1] Cox D.R. (1972). Regression models and life-tables. J Royal Stat Soc Series B (Methodological).

[2] Stensrud M.J., Aalen J.M., Aalen O.O., Valberg M. (2019). Limitations of hazard ratios in clinical trials. Eur Heart J.

[3] Kim D.H., Uno H., Wei L.J. (2017). Restricted mean survival time as a measure to interpret clinical trial results. JAMA Cardiol.

[4] Zhao L., Claggett B., Tian L. (2016). On the restricted mean survival time curve in survival analysis. Biometrics.

[5] Claggett B., Packer M., McMurray J.J. (2015). Estimating the long-term treatment benefits of sacubitril-valsartan. N Engl J Med.

[6] Royston P., Parmar M.K. (2013). Restricted mean survival time: an alternative to the hazard ratio for the design and analysis of randomized trials with a time-to-event outcome. BMC Med Res Methodol.

[7] Claggett B., Lachin J.M., Hantel S. (2018). Long-term benefit of empagliflozin on life expectancy in patients with type 2 diabetes mellitus and established cardiovascular disease. Circulation.

[8] Uno H., Claggett B., Tian L. (2014). Moving beyond the hazard ratio in quantifying the between-group difference in survival analysis. J Clin Oncol.

[9] Stienen S., Ferreira J.P., Vincent J. (2019). Estimated long-term survival with eplerenone. J Am Coll Cardiol.

