# Supplementary material for: Estimating the Lifetime Benefits of Treatments for Heart Failure
Source: JACC Heart Fail. 2020 Dec;8(12):984–95. doi: 10.1016/j.jchf.2020.08.004 (PMC7720789; doi:10.1016/j.jchf.2020.08.004)
Supplement: Supplemental Data [file mmc1.docx]

**Supplemental Material**

Supplemental Table 1. Treatment effect estimates in PARADIGM-HF by median NT-pro BNP

| **PARADIGM-HF** (Total n. =8399; Enalapril n. =4212; Sacubitril/Valsartan n. =4187) | | | | |
| --- | --- | --- | --- | --- |
| **CV death or HFH*** | **Enalapril** | **Sac./Valsartan** | **Treatment Effect** | **p-value** |
|  | | | | |
| **NT-pro BNP <Median (1615 pg/mL)** (N. =4195; Enalapril n. =2116; Sacubitril/Valsartan n. =2079) | | | | |
| Events and HR (95%CI) | 387 (18.2%) | 289 (13.9%) | 0.74 (0.63-0.86) | <0.001 |
| Event free survival (%) | 77.3 (75.1-79.4) | 82.7 (80.5-84.5) | +5.3 | <0.001 |
| Number Needed to Treat to benefit | | | 24 (16-48) | <0.001 |
| Event rates and difference (per 100py) | 8.8 (8.0-9.7) | 6.5 (5.8-7.3) | -2.3 (-3.5 to -1.2) | <0.001 |
| RMST (using follow-up time in days) | 959 (946-972) | 993 (982-1004) | +34 (17-51) | <0.001 |
| RMST (using age instead of follow-up time)****** | 9.2 (8.3-10.1) | 11.6 (10.6-12.6) | +2.4 (1.1-3.8) | 0.001 |
| **NT-pro BNP ≥Median (1615 pg/mL)** (N. =4190; Enalapril n. =2087; Sacubitril/Valsartan n. =2103) | | | | |
| Events and HR (95%CI) | 698 (33.4%) | 603 (28.7%) | 0.83 (0.75-0.93) | 0.001 |
| Event free survival (%) | 60.8 (58.3-63.3) | 64.6 (62.0-67.1) | +3.8 | 0.001 |
| Number Needed to Treat to benefit | | | 25 (16-61) | 0.001 |
| Event rates and difference (per 100py) | 18.4 (17.1-19.8) | 15.2 (14.1-16.5) | -3.1 (-4.9 to -1.3) | 0.001 |
| RMST (using follow-up time in days) | 821 (804-837) | 862 (846-877) | +41 (18-63) | <0.001 |
| RMST (using age instead of follow-up time)****** | 5.8 (5.1-6.5) | 6.7 (5.9-7.5) | 0.9 (-0.1 to 2.0) | 0.087 |

Legend: CVD, cardiovascular death; HFH, hospitalization for heart failure; HR, hazard ratio; CI, confidence interval; RMST, restricted mean survival time; NA, not applicable because the absolute risk reduction is not statistically significant.

*For consistency the analysis using follow-up time were capped at 3 years.

**For consistency the RMST using age instead of time used the same age range from 60 to 80 years in the studied subgroups of the PARADIGM-HF trial. This metric is expressed in years.

**Brief description of the included studies and respective outcomes**

1. The angiotensin–neprilysin inhibition versus enalapril in heart failure (PARADIGM-HF) trial^1^ enrolled 8442 patients with class ≥II heart failure and an ejection fraction ≤40% to receive either sacubitril/valsartan or enalapril. The primary outcome was a composite of heart failure hospitalization or cardiovascular death. We also studied all-cause death.
2. The effect of spironolactone on morbidity and mortality in patients with severe heart failure (RALES) trial^2^ enrolled 1663 patients with severe heart failure and an ejection fraction ≤35% to receive spironolactone or placebo. We studied the composite outcome of heart failure hospitalization or heart failure death and all-cause death.
3. The eplerenone in patients with systolic heart failure and mild symptoms (EMPHASIS-HF) trial^3^ enrolled 2737 patients with class II HF and an ejection fraction ≤35% to receive eplerenone or placebo. The primary outcome was a composite of cardiovascular death or heart failure hospitalization. We also studied all-cause death.
4. The Effect of Digoxin on Mortality and Morbidity in Patients with Heart Failure (DIG) trial^4^ was a multicenter, randomized, double-blind trial including patients with a left ventricular ejection fraction ≤45%, randomly assigned to digoxin (3397 patients) or placebo (3403 patients) in addition to diuretics and angiotensin-converting-enzyme inhibitors (median dose of digoxin, 0.25 mg per day; average follow-up, 37 months). We studied the composite outcome of heart failure hospitalization or heart failure death and all-cause death.

**References**

1. McMurray, J. J.; Packer, M.; Desai, A. S.; Gong, J.; Lefkowitz, M. P.; Rizkala, A. R.; Rouleau, J. L.; Shi, V. C.; Solomon, S. D.; Swedberg, K.; Zile, M. R., Angiotensin-neprilysin inhibition versus enalapril in heart failure. *N Engl J Med* **2014,** *371* (11), 993-1004.

2. Pitt, B.; Zannad, F.; Remme, W. J.; Cody, R.; Castaigne, A.; Perez, A.; Palensky, J.; Wittes, J., The effect of spironolactone on morbidity and mortality in patients with severe heart failure. Randomized Aldactone Evaluation Study Investigators. *N Engl J Med* **1999,** *341* (10), 709-17.

3. Zannad, F.; McMurray, J. J.; Krum, H.; van Veldhuisen, D. J.; Swedberg, K.; Shi, H.; Vincent, J.; Pocock, S. J.; Pitt, B., Eplerenone in patients with systolic heart failure and mild symptoms. *N Engl J Med* **2011,** *364* (1), 11-21.

4. The effect of digoxin on mortality and morbidity in patients with heart failure. *N Engl J Med* **1997,** *336* (8), 525-33.
